# Caring for COVID’s most vulnerable victims: a safety-net hospital responds

**DOI:** 10.21203/rs.3.rs-97328/v1

**Published:** 2020-10-26

**Authors:** Miriam Komaromy, Miriam Harris, Rob M. Koenig, Mary Tomanovich, Glorimar Ruiz-Mercado, Joshua A. Barocas

**Affiliations:** Grayken Center for Addiction, Section of General Internal Medicine, Boston Medical Center, Boston University; Grayken Center for Addiction, Section of General Internal Medicine, Boston Medical Center, Boston University; Boston Medical Center; Grayken Center for Addiction, Section of General Internal Medicine, Boston Medical Center; Grayken Center for Addiction, Section of Infectious Diseases, Boston Medical Center; Grayken Center for Addiction, Section of General Internal Medicine, Section of Infectious Diseases, Boston Medical Center, Boston University

**Keywords:** COVID-19, homelessness, substance use disorders, mental illness, hospital bed capacity

## Abstract

**Background:**

As COVID-19 surged in people experiencing homelessness, leaders at Boston Medical Center (BMC), New England’s largest safety-net hospital, developed a program to care for them.

**Aim:**

Provide an opportunity for COVID-infected people experiencing homelessness to isolate and receive care until no longer contagious

**Setting:**

A decommissioned hospital building.

**Participants:**

COVID-infected people experiencing homelessness

**Program Description:**

Care was provided by physician volunteers and furloughed staff. Care focused on allowing isolation, managing COVID-19 symptoms, harm-reduction interventions, and addressing problems related to substance use and mental illness.

**Program evaluation:**

Among 226 patients who received care, 65% were referred from BMC. Five percent were transferred to the hospital for a complication that appeared COVID-related. There were no deaths, but 7 patients had non-fatal overdoses. Seventy-nine *%* had at least one diagnosis of mental illness, and 42% reported actively using at least one substance at the time of admission. Thirty % had at least one mental health diagnosis plus active substance use.

**Discussion:**

This hospital-based COVID Recuperation Unit was rapidly deployed, provided safe isolation for 226 patients over 8 weeks, treated frequent SUD and mental illness, and helped prevent the hospital’s acute-care bed capacity from being overwhelmed during the peak of the COVID-19 epidemic.

## Background

People experiencing homelessness (PEH) are at increased risk of infection from COVID-19^1–3^, and recommended infection control measures are often not feasible^4^. Frequent handwashing is difficult, shelters are crowded, and physical distancing is impossible; beds often have no barriers between them and are located in large rooms. Personal protective equipment (PPE) may not be available for guests or shelter staff. When COVID-19 infection occurs in PEH, they are often unable to isolate at home, and may lack familial supports. These patients need help in order to recuperate, and it is essential that they isolate in order to reduce the risk of transmitting COVID-19 infection^5^.

PEH have higher rates of substance use disorders (SUDs) and mental health disorders than the general population^6^. SUDs pose special challenges during the COVID-19 epidemic^7^. Harm reduction approaches traditionally rely on in-person interactions (e.g. mobile outreach)^8^ to build relationships and disseminate supplies. The COVID-19 physical distancing mandate disrupts programs’ normal operations, risking greater incidence of complications like overdose. In isolation or quarantine settings, people with SUDs are at risk of withdrawal while confined without access to substances or treatment.

Boston Medical Center (BMC) has served as a safety net hospital in Boston since its founding in 1855. Approximately 9% of patients admitted to BMC are experiencing homelessness. As a result, BMC leaders prioritized addressing the unique needs of this vulnerable population.

## Aim

BMC created a COVID Recuperation Unit (CRU) site to provide a safe and supportive place for PEH to isolate and receive care for SUDs and mental health disorders. The CRU may provide a practical model for municipalities to provide care for PEH who are COVID-19 infected, and may inform future pandemic-planning efforts.

## Setting

The first Massachusetts case of COVID-19 was reported in Boston on February 1,2020^9^. In early March 2020, addiction and infectious disease specialists at BMC began conversations with BMC leadership about the need to create capacity for PEH with COVID-19 to isolate and receive care, even if they did not require hospitalization.

Clinical and strategy-team leaders began daily meetings with a group of organizations that provide care for PEH in Boston, including the Boston Public Health Commission, Department of Neighborhood Development, and Bureau of Recovery Services; harm reduction programs; and Boston Healthcare for the Homeless program, to forecast needs and develop alternative care sites.

In late-March, screening revealed high prevalence of COVID-19 infection in shelters. It became clear that larger facilities with expanded capacity were needed for PEH who were infected with COVID-19 in order to prevent rampant viral transmission. Within BMC, clinical leaders raised reasonable concerns about diverting workforce from staffing inpatient and intensive care units during the predicted surge in COVID-19 hospitalizations. However, the hospital CEO advocated for BMC addressing this public health need in Boston. Hospital leadership were also unified around a goal of preserving BMC’s ability to serve the community through the crisis. Like many hospitals, BMC expected to reach maximum bed capacity, and leadership was motivated to identify alternate care sites for patients who needed isolation but did not need to occupy inpatient beds. BMC leadership asked the Commonwealth of Massachusetts to loan BMC a vacant hospital building near the BMC campus for the development of a COVID Recuperation Unit (CRU) to serve PEH. On March 24, 2020, state leaders agreed.

## Participants

The CRU served patients who were COVID-infected and experiencing homelessness.

## Program Description

### Rapid deployment was assisted by crisis status

The CRU admitted its first patients on April 9, 2020. Between March 24 and April 9, several features of the crisis allowed for rapid implementation of the CRU. First, the Massachusetts Department of Public Health (DPH) licensure for the CRU was obtained under the *Authorization and Guidelines for Use of Alternate Space for Treatment of Patients During the COVID19 2020 State of Emergency*^10^ issued by the DPH pursuant to the Commonwealth’s Emergency Declaration. This allowed the hospital to perform clinical care in unlicensed space not owned by BMC. Second, the CRU was classified as a medicalized shelter by DPH, and as a “bedded outpatient” unit by the Drug Enforcement Administration (DEA), avoiding the need to qualify for inpatient level of care and allowing for medications to be prescribed on an outpatient basis. This, in turn, allowed the CRU to operate without an inpatient pharmacy. Third, anticipation of emergency state and federal funding allowed for simplified processes and documentation since billing was not required. Fourth, staffing was available in part because the health system paused non-essential services. BMC medical staff were available to work in the CRU because research activities and many clinical and educational programs were suspended. Fifth, the CRU was able to utilize many of BMC’s existing systems and relationships. Clinical programs in BMC’s Grayken Center for Addiction, such as harm reduction, addiction treatment programs, and counseling/social work came together to support clinical services. The CRU also leveraged BMC’s preparations for the COVID-19 crisis, such as supplies of PPE, support from Facilities Management, IT, infection control, and Admitting. In addition, BMC’s Development department received donations for the CRU including furniture, televisions, clothing, and prepackaged meals delivered three times per day for patients and staff.

### Description of the COVID Recuperation Unit

All staff wore full PPE at all times. Because all patients were COVID-infected, patients were not required to wear PPE. The unit was staffed 24/7 (Table 1). While acute exacerbations of non-COVID-related medical or mental health issues were addressed, there was less focus on managing chronic health problems. Patients self-administered most medications, which they kept locked at their bedside.

Many patients who were admitted to the CRU had SUDs. While treatment was offered, it was not expected that all patients who used substances would be interested in engaging in treatment. Several aspects of the COVID-19 epidemic drove adaptations to SUD care:

1. Frequent withdrawal: Withdrawal was common because patients were confined suddenly. All patients were assessed for withdrawal risk upon admission, and medical treatment of withdrawal was available 24 hours per day.
2. Addiction Consultation: Some medical staff were not comfortable managing SUDs, and because of changes in regulations regarding telehealth, addiction specialists at BMC were able to perform telehealth consults, including managing buprenorphine and methadone induction.
3. Methadone treatment: Initially, methadone was obtained via take-home doses for patients who were already enrolled in outside opioid treatment programs (OTPs). This was operationally challenging, and some patients with opioid withdrawal were not enrolled in OTPs, but buprenorphine was contraindicated (if, for instance, they had been using illicitly-obtained methadone or longer-acting fentanyl analogs); or if methadone was not their preferred pharmacologic treatment. Therefore, medical staff consulted with the DEA and obtained the ability to start methadone on-site.
4. Harm Reduction: A harm reduction philosophy was especially important because patients were typically not seeking treatment for SUDs when they were admitted. Harm reduction specialists were onsite for support and staff education, and for provision of Naloxone and rapid HIV tests. Safe injection supplies were offered to patients at the time of discharge.
5. Goal of SUD management: In this setting the overarching goal of medical management for SUDs was to help patients to tolerate isolation and quarantine. At times this necessitated adaptations of usual practice. For instance, whereas the goal of medication treatment for OUD might usually be to avoid intoxication or withdrawal, in this setting minor intoxication was accepted according to individual patient needs and preferences. Additionally, addiction specialists could offer stimulant or benzodiazepine prescriptions for severe stimulant or benzodiazepine use disorders to reduce discomfort, decrease cravings, and reduce the need to leave the unit for substance use. Cigarettes, usually banned from hospital campuses, were provided to patients upon request, and smoking areas were designated in a courtyard outside the building.

## Program Evaluation

Admissions to the CRU were tracked in the BMC hospital admissions system and in the Epic Electronic Health Record. A retrospective analysis of these data was performed after the CRU closed on June 4^th^, 2020, including source of referrals, patient demographics, presence of COVID-19 symptoms, co-occurring mental health or substance use problems, length of stay, whether patients had acute complications, transfers to acute care settings, and clearance from isolation precautions. Reported data were based on simple counts via clinical chart review. This study was approved by the Boston University Medical Campus Institutional Review Board (H-40286).

Between April 9 and June 4, 2020, 226 patients were treated in the CRU, with an average length of stay of 7.3 days. Most (146) were referred from BMC impatient or emergency department (ED), and the rest from other hospitals, shelters, and testing sites. Seventy-two percent were male; 39% identified as Black, 11% Hispanic/ Latinx (Table 2). Seventy-nine percent had ≥ 1 psychiatric diagnosis, and 42% reported active substance use (Table 3). There were no deaths. At least 7 patients experienced a non-fatal overdose and 5% of patients developed serious complications of COVID-19 (Table 4). Seven percent of patients left prior to being medically cleared from isolation, but 1/3 of those who left AMA subsequently returned. After completing isolation, 24 patients were discharged to SUD or mental health programs, 28 to stay with family members, and the rest to shelters (data not shown).

The CRU preserved inpatient beds for patients who needed acute care in at least two ways.^11^ First, the CRU admitted patients who did not need acute care but could not be discharged from the ED due to risk of contagion. Second, the CRU admitted patients who were medically ready for discharge after a hospitalization but still needed isolation.

## Discussion

### Challenges

Although the development and implementation of the CRU was generally quite successful, the program faced a number of challenges.

Staffing: Staff were recruited from various settings and did not work full time on the unit. Many did not have experience working with PEH or managing mental illness and substance use. This made it difficult to create a consistent culture of harm-reduction free of stigma in the CRU.

Determining appropriate discharge criteria: Criteria for clearance from isolation precautions changed frequently due to evolving CDC recommendations and shortage of COVID tests. Swab-based discharge criteria turned out to be impractical, and inconsistent with the approach taken by partner organizations, since many patients’ swabs remained positive for weeks; and so discharge criteria were revised to align with the CDC’s 10-day symptom-based discharge^12^.

Predicting demand for beds: Demand was driven by the curve of the epidemic, but also by two other factors. First, COVID-19 testing was intermittent in PEH. When testing occurred, large groups of patients were suddenly identified who needed admission for isolation and quarantine^4^. A second challenge was the tremendous variability in the prevalence of COVID-19 infection that was found during testing, ranging from 37% to 0% over the course of 8 weeks. This variability meant that daily admissions ranged from 0 to 17, making it difficult to predict staffing needs.

Patient use of drugs/harm reduction: The Commonwealth of Massachusetts operated isolation and recovery units, typically set up at hotels, in other parts of the state to provide housing for COVID-infected PEH. Guidance documents for staff promoted a harm reduction approach. These units collaborated with harm reduction agencies which provided harm reduction materials, including sterile syringes and naloxone rescue kits. People who used drugs or alcohol were not discharged for substance use. Staff worked with guests to stay safe (personal communication, Dr. Alex Walley, Medical Director for Overdose Prevention Program, MA Dept. of Public Health). Within the CRU, it was challenging to fully implement a harm-reduction approach. There was disagreement over the appropriate approach, with many medical staff advocating for harm-reduction, while security personnel as well as some staff and administration officials expressed concerns regarding safety on hospital grounds and public perception regarding laws that prohibit supervised injection sites. A compromise included distribution of sterile syringes at the time of discharge (rather than admission), and a policy of having clinicians, rather than security personnel, address use of substances.

Caring for patients with serious mental illness: Serious mental illness was common, and initially staff were uncomfortable caring for patients who were paranoid, actively hallucinating, or delusional. Later, counselors and social workers became available to assist on the unit, which lessened staff concerns. The CRU filled an important need in the community by providing care for COVID-infected patients with serious mental illness^13^.

### Lessons learned

BMC expanded its role as a safety-net hospital to provide care for COVID-infected PEH, and utilized the opportunity to initiate treatment for SUDs for many patients, as well as caring for patients with severe psychiatric disorders. Rapid deployment of services in this emergency was achieved through hospital and Commonwealth coordination, and relaxation of regulations to allow speed and efficiency. Community partnerships were key factors in our success.

The CRU helped BMC avoid exceeding hospital bed capacity during the epidemic surge^11^. Lower-acuity bed capacity in the CRU provided a vitally-important release mechanism to allow BMC to reserve inpatient beds for patients with critical needs. Other cities that are currently being affected by the COVID-19 surge should consider similar programs that increase lower-acuity bed capacity for vulnerable populations.

Finally, the experience of creating the CRU has shown us that we can provide safe and effective medical respite for PEH and has inspired us to explore the implementation of similar services beyond the COVID-19 pandemic.

## Figures and Tables

**Table 1.** Staffing Model at COVID Recuperation Unit

Fixed staffing, 2 floors	Day FTE	Night FTE
MD	1.00	1.00
Operations director	1.00	1.00
Discharge planner	1.00	n/a
Security staff	4.00	4.00

Variable staffing ratio, per patient	Day	Night
NP/PA	1:88	n/a
RN/LPN	1:33	1:33
BH provider (counselor, social worker)	1:88	n/a
Harm reductionist	1:88	n/a
Nurse Tech/MA	1:22	1:22
Operations staff	1:22	1:22

**Table 2.** Patient characteristics during treatment at COVID Recuperation Unit, Massachusetts 2020 (n=226)

Baseline Characteristics	Full Cohort
	n (%)
Race/ethnicity	
Black, Non-Hispanic	88 (38.9%)
White, Non-Hispanic	71 (31.4%)
Hispanic or Latinx	24 (10.6%)
Other	3 (1.4%)
Unknown	40 (17.7%)
Psychiatric Comorbidities*^+^	
Depression	86 (38.1%)
Anxiety	78 (34.5%)
PTSD	42 (18.6%)
Bipolar Disorder	37 (16.4%)
Schizophrenia/Schizoaffective Disorder	26 (11.5%)
Brain Injury/TBI	13 (5.8%)
Active Substance Use at Time of Admission*^+^	
Alcohol	64 (28.3%)
Opioids	43 (19.0%)
Cocaine/Crack	43 (19.0%)
Methamphetamines	4 (1.8%)
Benzodiazepines (not prescribed)	3 (1.3%)
	

* not mutually exclusive

+ reported in electronic health record or medical provider’s assessment

**Table 3.** Baseline psychiatric diagnoses and active substance use status in COVID Recuperation Unit, Massachusetts 2020 (n=226)

Baseline diagnoses	Full Cohort n (%)
Patients with psychiatric diagnoses	179 (79.2%)
Patients with two or more psychiatric diagnoses	86 (38.0%)
Patients with active drug use*	94 (41.6%)
Patients who actively use more than one drug	40 (17.7%)
Patients who have at least one psychiatric diagnosis and actively use at least one substance*	68 (30.1%)

* excludes tobacco and marijuana use

**Table 4.** Discharge/transfer events to Boston Medical Center for medical/psychiatric complications from COVID Recuperation Unit, Massachusetts 2020 (n=226)

Rationale for evaluation	Full Cohort n (%)
Medical evaluation - apparent exacerbation of COVID-19 symptoms	11 (4.9%)
Acute respiratory failure and/or low oxygen saturation	5 (2.2%)
Acute cardiac issues	4 (1.8%)
Coagulation issues	1 (0.4%)
Renal issues	1 (0.4%)
Medical evaluation - apparently unrelated to COVID-19 infection	9 (4.0%)
Psychiatric evaluation	7 (3.1 %)
